# An update on our understanding of Gram-positive bacterial membrane vesicles: discovery, functions, and applications

**DOI:** 10.3389/fcimb.2023.1273813

**Published:** 2023-10-04

**Authors:** Yiyun Xu, Chonghong Xie, Yong Liu, Xiaosong Qin, Jianhua Liu

**Affiliations:** Department of Laboratory Medicine, Shengjing Hospital of China Medical University, Liaoning Clinical Research Center for Laboratory Medicine, Shenyang, China

**Keywords:** Gram-positive bacteria, membrane vesicles, function, application, virulence

## Abstract

Extracellular vesicles (EVs) are nano-sized particles released from cells into the extracellular environment, and are separated from eukaryotic cells, bacteria, and other organisms with cellular structures. EVs alter cell communication by delivering their contents and performing various functions depending on their cargo and release into certain environments or other cells. The cell walls of Gram-positive bacteria have a thick peptidoglycan layer and were previously thought to be unable to produce EVs. However, recent studies have demonstrated that Gram-positive bacterial EVs are crucial for health and disease. In this review, we have summarized the formation, composition, and characteristics of the contents, resistance to external stress, participation in immune regulation, and other functions of Gram-positive bacterial EVs, as well as their application in clinical diagnosis and treatment, to provide a new perspective to further our understanding of Gram-positive bacterial EVs.

## 1 Introduction

Extracellular vesicles (EVs) are nanoparticles surrounded by a lipid bilayer, secreted by bacteria or eukaryotic cells (Derkus et al., 2017). In 1966, EVs from *E. coli* were first observed by Work et al. and described as cellular vesicles containing lipopolysaccharides and interstitial membrane proteins secreted by outer membrane protrusions (Work et al., 1966). Subsequently, Gram-negative bacteria, such as *Pseudomonas aeruginosa*, *Salmonella* Typhimurium, and *Helicobacter pylori*, have been shown to produce and secrete EVs (Mullaney et al., 2009; Bai et al., 2014; Sartorio et al., 2021).

EVs are also known as outer membrane vesicles (OMVs) in Gram-negative bacteria and membrane vesicles (MVs) in Gram-positive bacteria. Bacterial EVs have various biological functions, such as exerting effects on bacterial virulence, antibiotic resistance, transfer of virulence genes, helping bacteria survive and evade immune responses, transporting biomolecules to host cells, and assisting in clinical disease treatment (Yaron et al., 2000; Kulp and Kuehn, 2010; Aung et al., 2016; Aytar Çelik et al., 2022). The study of EVs is continuously increasing; however, most studies are currently focused on eukaryotic and Gram-negative bacteria, and studies on EVs in Gram-positive bacteria remain limited.

Gram-positive bacteria, especially Gram-positive cocci, such as coagulase-negative *Staphylococcus, Staphylococcus aureus, Enterococcus*, and *Clostridium difficile*, are crucial pathogens in hospitals. According to the 2021 China Antimicrobial Surveillance Network (CHINET) data (Hu et al., 2022), Gram-negative bacteria accounted for 71.4% of the clinical isolates from member hospitals, whereas Gram-positive bacteria accounted for 28.6%, which is approximately half the number of Gram-negative bacterial cases. Although the number of cases is not as high as that of Gram-negative bacteria, they can cause severe infections and increase mortality. In 2016, the number of deaths in 195 countries owing to *Streptococcus pneumoniae*-induced lower respiratory tract infections alone was approximately 1.19 million (Troeger et al., 2018).

With the widespread use of antibiotics, drug-resistant bacteria have evolved, and the number of antibiotic-resistant Gram-positive bacteria strains has increased in some countries (Lessa et al., 2015; Lakhundi and Zhang, 2018; Watkins et al., 2022). Multi-drug resistant (MDR) bacteria, such as methicillin-resistant *Staphylococcus aureus* (MRSA), vancomycin-resistant *Enterococcus faecalis* (VRE), and β-lactamase-producing *Streptococcus pneumoniae*, often render multiple antibiotic treatments ineffective, resulting in difficult-to-cure chronic infection in patients, and an increased mortality rate. According to the statistics, the in-hospital mortality rate of bloodstream infections caused by *Staphylococcus aureus* is approximately 32.4% in middle-income countries (Bai et al., 2022). Despite a decrease in the mortality rate over the past 30 years, at least 25% of patients die within three months (Bai et al., 2022).

In this context, MVs are vital in bacterial virulence factor pathogenesis, drug resistance mechanisms, and immune regulation. Therefore, the study of Gram-positive bacterial MVs is of considerable significance in gaining insights into the pathogenesis of Gram-positive bacteria and providing novel therapeutic approaches for clinical practice. Therefore, in this paper, we review the composition and characteristics of Gram-positive bacterial MVs, their functions in auxiliary escape and immunomodulation, and their clinical applications.

## 2 Formation of Gram-positive bacteria MVs

In Gram-negative bacteria, there are three main hypotheses for vesicle formation: membrane curvature-inducing proteins induce the formation of spherical vesicles by increasing the membrane curvature, dissociation of the crosslinking between the outer membrane and peptidoglycan to produce vesicles, and mutations in the VacJ/Yrb ATP-binding box (ABC) transport system inducing vesicle production (Roier et al., 2016; Toyofuku et al., 2019). Gram-positive bacteria have not been studied as extensively as Gram-negative bacteria, and we lack a complete model for the formation of MVs. It is now believed that the formation of Gram-positive bacterial MVs is primarily mediated by the outgrowth of specific lipid-enriched regions within the cytoplasmic membrane, which subsequently weakens the thick peptidoglycan layer in the presence of endolysins and facilitates MV release (Toyofuku et al., 2019). Furthermore, the formation of MVs is not determined by a small number of genes but is regulated by a complex network of genes (Briaud and Carroll, 2020).

MVs are produced by various Gram-positive bacteria, including *Staphylococcus aureus, Streptococcus pneumoniae, Mycobacterium tuberculosis, Lactobacillus, Bacillus subtilis, Bacillus anthracis, Listeria monocytogenes*, and *Clostridium perfringens* (Jiang et al., 2014; White et al., 2018; Choi et al., 2019; Karthikeyan et al., 2020; Abe et al., 2021; Bitto et al., 2021; Mehanny et al., 2022). Their diameters range between 20 and 500 nm (Brown et al., 2015). The differences in the size of MVs may be related to the purification technique and measurement method used (Aytar Çelik et al., 2022), environmental factors of vesicle growth, and the influence of membrane lipid genes (Toyofuku et al., 2019).

For example, Codemo et al. observed *Streptococcus pneumoniae* MVs in the range of 25–250 nm by electron microscopy using the OptiPrep density gradient media method (Codemo et al., 2018), whereas Mehanny et al. determined MVs in the range of 130–160 nm using size exclusion chromatography (SEC) for vesicle purification and nanoparticle tracking analysis (NTA) (Mehanny et al., 2020). The sizes of bacterial MVs also vary across different growth periods (Mehanny et al., 2022). Bacteria must uptake and utilize nutrients from the environment during growth. The production of EVs is influenced by many environmental factors, such as hypoxia, temperature, iron deficiency, and antibiotic stimulation (Orench-Rivera and Kuehn, 2016). Poor external environmental conditions such as non-growing temperatures and high salt, acid, and iron deficiencies increase the secretion of MVs (Lee T. et al., 2017; Karthikeyan et al., 2020; Wang et al., 2021).

## 3 Composition and characteristics of Gram-positive bacteria MVs

**Figure 1:**
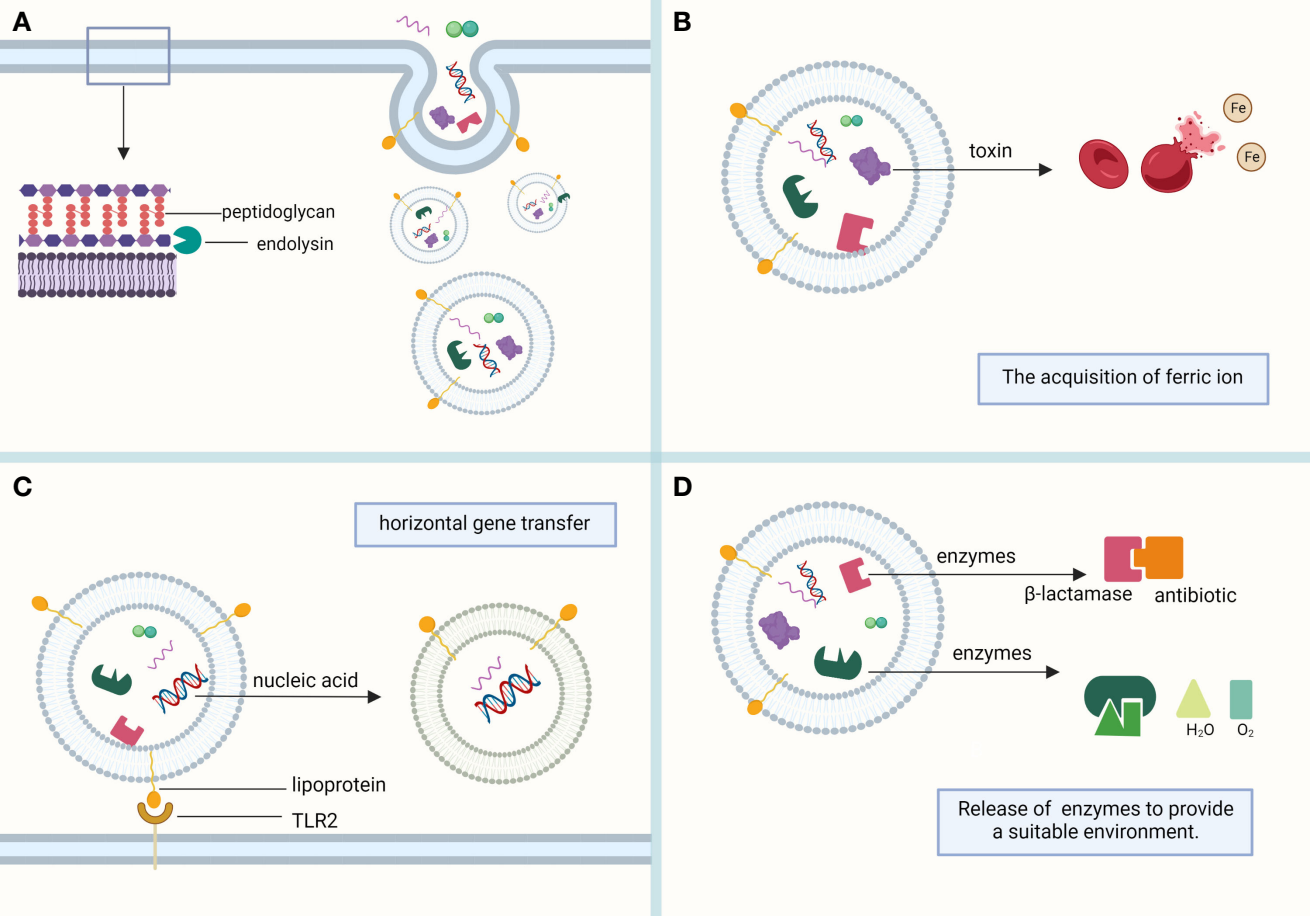
Composition of MVs. **(A)** MVs sprout in the lipid-rich region of the cell membrane and are formed with the help of the endolysin, which weakens the bacterial peptidoglycan layer. MVs mainly contain phospholipids, proteins, and nucleic acids, which play their respective roles. **(B)** Toxins in MVs, such as alpha-hemolysin and leukocidins, help lyse red blood cells to obtain the iron required for bacterial survival. **(C)** Bacterial heritable materials can affect the biological function of bacteria through MV-mediated horizontal gene transfer. Lipoprotein, a membrane protein contained in MVs, is involved in immune response as a Toll-like receptor 2 (TLR2) ligand. **(D)** The antibiotic-inactivating enzymes and catalases contained in MVs can hydrolyze antibiotics and reactive oxygen species, respectively, to provide a suitable living environment for bacteria. (This figure was created using Biorender.com).

EVs of Gram-positive bacteria contain fatty acids, phospholipids, cytoplasmic proteins, membrane-associated virulence proteins, lipid acids, peptidoglycans, DNA, and sRNA (Yu et al., 2018) (**Figure 1**).

### 3.1 Proteins

The proteins in bacterial EVs perform various physiological functions. Gram-negative and Gram-positive bacteria both secrete proteins, but the protein composition and quantity within bacterial EVs vary. Notably, the types of proteins detected in the MVs of different strains vary significantly within the same genus (Olaya-Abril et al., 2014; Mehanny et al., 2022). For example, Jeon et al. reported *via* proteomic analysis that MVs isolated from three *S. aureus* strains from clinical sources contained 60–85 specific proteins with varying cytotoxicities (Jeon et al., 2016). Nonetheless, core proteins, such as the surface protein PspA, ABC transporter protein substrate-binding protein, and penicillin-binding protein 1 B, are mostly conserved.

Lipoprotein enrichment has also been observed in the proteomic analysis of MVs of most Gram-positive bacteria, such as *S. aureus, S. pneumoniae, S. pyogenes, B. subtilis, and M. tuberculosis* (Lee et al., 2009; Brown et al., 2014; Olaya-Abril et al., 2014; Biagini et al., 2015; Lee et al., 2015). Bacterial lipoproteins are a group of membrane proteins with many functions and are key ligands of Toll-like receptor 2 (TLR2) in Gram-positive bacteria that play a vital role in host immune responses to bacterial infection (Zähringer et al., 2008; Tomlinson et al., 2014). Lipoprotein deficiency affects bacterial growth, immune activation, and virulence (Stoll et al., 2005; Khandavilli et al., 2008). The absence of pre-prolipoprotein diacylglyceryl transferase (Lgt) in *S. pneumoniae* delays bacterial growth and reduces its invasion capacity (Chimalapati et al., 2012).

Furthermore, lipoproteins influence the MVs they produced. The absence of lipoproteins significantly affects the host immune response to *S. pneumoniae* MVs. MVs produced by lipoprotein-deficient *S. pneumoniae* mutants lead to decreased nuclear factor-kappa B (NF-κB) activity and reduced cytokine production by macrophages in mice, and the levels of immune responses induced by these cytokines are reduced (Yerneni et al., 2021). Similarly, the immunostimulatory ability of MVs produced by Lgt-mutant *S. aureus* in mice decreased significantly (Kopparapu et al., 2021). In *M. tuberculosis* MVs, lipoproteins stimulate the TNF-α production by naïve macrophages (Bhatnagar et al., 2007). Thus, lipoproteins are crucial in regulating systemic immune responses to MVs.

### 3.2 Virulence factors and toxins

The production of virulence factors or toxins is a common survival and attack mechanism of microorganisms, which is necessary for host pathogenesis (Weiser et al., 2018) and is a crucial component of MVs. Pathogenic Gram-negative bacterial vesicles can encapsulate these virulence factors for delivery to host cells (Bomberger et al., 2009), and a similar phenomenon can also be observed in Gram-positive bacteria.

Virulence factors vary among different bacterial MVs and play multiple roles in regulating host responses to bacterial infection and contribute to the colonization and invasion of bacteria (**Table 1**). The adhesins CapD and PrpA, and collagen-binding proteins Acm and Scm, detected in *Enterococcus faecium* MVs contribute to the adhesion and colonization of *E. faecium* (Wagner et al., 2018). Virulence factors, such as α-hemolysin contained in *S. aureus* MVs, promote the survival of *S. aureus* in human blood while conferring bacterial resistance to neutrophils (Askarian et al., 2018). *Propionibacterium acnes* MVs contain hyaluronate lyase, a critical virulence factor that invades host cells (Jeon et al., 2016). Additionally, bacteria can promote infection and induce immunomodulatory responses after delivering virulence factors to the host cells through EVs. *B. anthracis* MVs contain a variety of toxins, such as protective antigen (PA), lethal factor (LF), edema factor (EF), and anthrolysin (ALO), which immunize mice. The PA causes mice to produce a strong IgM response to the toxin and deliver the toxin to host cells, increasing the potency of the toxin (Rivera et al., 2010). *Listeria monocytogenes* MVs contain a toxic force protein, listeriolysin O, which induces increased cytotoxicity in the human colon adenocarcinoma Caco-2 cell line, contributes to internalization and participates in interactions with host proteins during bacterial infections (Karthikeyan et al., 2019; Karthikeyan et al., 2020). Toxins in MVs are transported to host cells to elicit their effects and exacerbate damage to the host. For example, staphylococcal protein A (SPA) in *S. aureus* MVs induces more severe eczematous dermatitis in patients with atopic dermatitis, which may be the result of SPA-induced production of pro-inflammatory cytokines and chemokines, leading to the recruitment of inflammatory cells and local inflammation in atopic dermatitis lesions (Jun et al., 2017).

**Table 1:** Virulence factors in vesicles of common clinical pathogenic bacteria.

Bacterium	Virulence factors	Reference
***Staphylococcus aureus***	α-Hemolysin,δ-hemolysin, Autolysin, SPA, ETA, ETC, and LukD	(Oogai et al., 2011; Jeon et al., 2016; Askarian et al., 2018)
***Streptococcus pneumoniae***	PLY, LPxTG Proteins, Cbp D/E/F, Lipoproteins, Autolysin, NanA/B/C, PsaA,PspA/C,etc	(Olaya-Abril et al., 2014; Codemo et al., 2018; Mehanny et al., 2022)
***Streptococcus suis***	SspA, SsnA, Ide, Plr, HtpsC, and Mrp etc.	(Haas and Grenier, 2015)
***Streptococcus pyogenes***	M protein, ScpA, streptolysin O, and Lipoproteins	(Biagini et al., 2015; Resch et al., 2016)
***Listeria monocytogenes***	LLO, internalin proteins, Autolysin, P60, PLC-A, FlaA	(Karthikeyan et al., 2019; Karthikeyan et al., 2020)
***Mycobacterium tuberculosis***	lipoproteins (such as LpqH, LprA, LprG), HbhA, TatA, and SodB	(Prados-Rosales et al., 2011; Lee et al., 2015)
***Bacillus anthracis***	PA, LF, EF, and ALO	(Rivera et al., 2010)
***Enterococcus faecium***	CapD, PrpA, Acm, Scm, AtlA, CcpA, VanA, SdrD, and Esp	(Wagner et al., 2018; Kim et al., 2019)

### 3.3 Genetic material

Dorward and Garon. (1989) discovered DNA in the vesicles of *Neisseria gonorrhoeae* (Dorward and Garon, 1989). With the continuous development of vesicle studies, RNA, including sRNA, mRNA, tRNA, and rRNA, has been discovered in the vesicles of Gram-negative bacteria (Quek et al., 2016; Han et al., 2019; Langlete et al., 2019; Diallo et al., 2022). Similar to bacteria, EVs carry genetic material that plays a vital role in their function. In addition to stabilizing and protecting transported proteins from hydrolytic digestion by extracellular proteases, bacterial EVs also protect their nucleic acid cargo from degradation by extracellular nucleases. Therefore, they can deliver numerous bioactive macromolecules to bacterially infected host cells (Doré and Boilard, 2023).

Gram-positive bacterial MVs also possess genetic material (Jiang et al., 2014; Surve et al., 2016; Domínguez Rubio et al., 2017) that can be safely translocated to host cells. For example, miRNAs in *Streptococcus sanguinis* MVs are protected from degradation by RNase A, and are thus safely transported to host cells (Choi et al., 2016). Group A streptococcal MVs contain RNAs that differ in abundance from the original bacteria, where the release of rRNA and tRNA is influenced by the two-component “control of virulence regulator-sensor” operon (covRS) (Resch et al., 2016).

Furthermore, encapsulated nucleic acid molecules participate in host cell immune responses through different pathways. The DNA and RNA detected in *S. aureus* MVs can be transported with MVs to host epithelial cells, thereby activating pattern recognition receptors (PRRs) to promote the release of cytokines and chemokines from epithelial cells, followed by autophagy-mediated degradation (Bitto et al., 2021). Transcriptomic sequencing data revealed that *Listeria monocytogenes* MVs contained various forms of RNA, including tRNA, rRNA, mRNA, and sRNA. rli32 sRNA in MVs correlated with the IFN-β response after incubation with bone marrow macrophages (Frantz et al., 2019). Despite the role of genetic material in Gram-positive bacterial MVs in immune regulation, the exact mechanisms by which these DNA and RNA molecules are involved in host cell immune regulation, and other mechanisms remain unclear.

## 4 Function of Gram-positive bacteria MVs

OMVs and MVs can be coated with proteins, toxins, nucleic acids, lipids, and other substances for transport to other bacterial species or mammalian cell receptors for their corresponding functions. They are critical for generating immune effects, mediating horizontal gene transfer, generating antimicrobial resistance, nutrient uptake, and transporting virulence factors. They are essential for the survival of both Gram-negative and Gram-positive bacteria. The functions of MVs are heavily mediated by their cargo (**Figure 1**).

### 4.1 Strengthening resistance to external pressure

EVs can enhance bacterial resistance to environmental factors, prevent bacterial damage by nutrient uptake from the environment, promote biofilm formation, and produce catalases and antibiotic-inactivating enzymes (**Figure 1**).

Iron is essential for bacterial survival and growth (Ferreira et al., 2016). Mycobactins are high-affinity iron chelators or siderophores secreted by *M. tuberculosis*. Iron deficiency results in increased MV production in *M. tuberculosis* and elevated local concentrations of mycobactins (as part of vesicles), which are subsequently released to provide extracellular iron to support bacterial proliferation (Prados-Rosales et al., 2011). The production of MVs from *S. aureus* increases under iron-deficient conditions in culture, and α-hemolysin, leukocidin LukED and HlgAB in MVs lyse erythrocytes to release hemoglobin and heme to promote iron acquisition by bacteria (Wang et al., 2021). Similarly, the presence of iron-binding factors in *Streptomyces coelicolor* contributes to bacterial survival under iron-limited conditions (Schrempf et al., 2011).

Bacterial EVs contain several substances that help bacteria survive external environmental stresses. Antibiotics are the most common environmental stressors encountered by bacteria. Antibiotic-inactivating enzymes can be released into EVs to degrade antibiotics, thereby counteracting the associated damage. MRSA produces MVs that increase in a dose-dependent manner in the presence of ampicillin and contain more β-lactamase enzymes and metallo β-lactamase superfamily proteins, which hydrolyze β-lactam antibiotics. These vesicle-encapsulated proteins can be transported over long distances to prevent degradation and significantly contribute to the development of antibiotic resistance in bacteria (Kim S. et al., 2020). MRSA can also transfer β-lactamases to antimicrobial‐sensitive *Escherichia coli via* MVs, causing them to secrete several times more β-lactamases than the parental strain to protect against antibiotic pressure (Lee et al., 2022). The expanding application of antibiotics and their excessive use expose bacteria to external stresses more frequently. Further investigation is required to ascertain whether EVs can be stably inherited as a protective mechanism against antibiotics, as well as whether such a mechanism exists in all bacteria.

In addition to producing antibiotic-inactivating enzymes, some MVs harbor substances, such as catalase, to protect bacteria from the disruptive effects of reactive oxygen species. For example, adding MVs to the growth medium of *L. monocytogenes* induces the production of more MVs under oxidative stress. Superoxide dismutase decomposition and catalase can be detected in these MVs, which may provide a suitable environment for bacteria in oxidative environments (Wang et al., 2021).

### 4.2 Participation in immune regulation

Immunomodulation is pivotal for the host response to bacterial infections. In addition to the direct interaction between bacteria and host immune cells, MVs released by bacteria can also contribute to host defense by directly or indirectly initiating immune responses and affecting immune cell populations, consequently triggering the recruitment of immune cells and release of pro-inflammatory cytokines.

MVs induce an immune response and participate in innate and adaptive immunity in Gram-positive bacteria. Among the innate immune responses stimulated by MVs in the host, the primary responses involve immune cells, such as macrophages and dendritic cells, as well as TLR molecules, and produce various pro-inflammatory factors. *S. pneumoniae* MVs induce NF-κB activation in a dose-dependent manner after co-incubation with macrophages, resulting in a significant increase in macrophages in the blood of mice injected with MVs (Yerneni et al., 2021). A similar phenomenon was observed in MVs secreted by *Streptococcus suis*, which may contribute to increased blood-brain barrier permeability (Haas and Grenier, 2015). *M. tuberculosis* MVs carry lipoproteins that are essential ligands for TLR2 and activate macrophage responses upon binding with TLR2 (Prados-Rosales et al., 2011).

Furthermore, MVs can mediate different infection outcomes through different intracellular pathways. MVs secreted during *Listeria monocytogenes* infection can accumulate in lysosomes through endocytosis in non-phagocytic cells and cause an autophagic response by releasing MVs from cells in the phagocytic body (Vdovikova et al., 2017). NOD-, LRR- and pyrin domain-containing 3 (NLRP3) inflammasome plays a vital role in the innate immune response and disease development. Its activation is a host defense mechanism for clearing damaged and infected cells. MVs released by *S. aureus* mediate the immune response of macrophages through the TLR2 signaling pathway and induce NLRP3 inflammasome activation and production of IL-1β and IL-18 (Wang et al., 2020), which contribute to the establishment of an effective innate immune response to *S. aureus* infection. Gram-positive bacterial MVs are also involved in adaptive immune responses, primarily through the stimulation of host antibody production, thereby providing immune protection. This property aids vaccines development.

### 4.3 Assisting bacterial survival and escape

Bacteria typically defend themselves against the immune response of host cells upon entry by producing MVs that release specific components and create a favorable environment for immune escape. For example, lipoglycans from MVs of *M. tuberculosis* are transported to T cells to stimulate the expression of GRAIL, a marker of T cell anergy, in CD4 + T cells, thus inhibiting T cell responses to facilitate immune escape (Athman et al., 2017). *S. pneumoniae* MVs can bind to complement C3 in the presence of choline-binding proteins, forming an attack membrane complex that reduces bacterial interactions with the complementary site on phagocytes and contributes to bacterial evasion of host humoral immunity (Codemo et al., 2018).

Neutrophil extracellular traps (NETs), produced after neutrophil death, are another response to bacterial elimination. NETs are DNA networks covered with antimicrobial peptides and histones that can degrade virulence factors and kill bacteria, and belong to a novel intrinsic immune defense mechanism (Brinkmann et al., 2004). During an immune attack by the host, DNase is produced to degrade NETs, which aids bacteria in escaping host killing. The DNase TatD contained in *S. pneumoniae* MVs helps the bacteria escape NETs; consequently, *S. pneumoniae* that lack TatD have diminished virulence (Jhelum et al., 2018). A similar phenomenon was observed in *S. suis* MVs (Haas and Grenier, 2015).

Simultaneously, MVs can help bacteria to resist killing within the host. Various toxins contained in *S. aureus* MVs increase the resistance of bacteria to neutrophils in human blood and promote bacterial survival (Askarian et al., 2018). MVs evade the killing effect of neutrophils in the host as well as act as molecular decoys to protect bacteria in some cases. To protect human skin and nasal secretions from antimicrobial fatty acids (AFAs), *S. aureus* releases MVs as decoys to bind to AFA and avoid damage caused by the combination of the bacterial membrane and AFAs (Kengmo Tchoupa and Peschel, 2020).

### 4.4 Horizontal gene transfer

Microorganisms transfer genetic material *via* horizontal gene transfer (HGT), which alters or influences biological functions. EVs contain various components, including DNA and various types of RNA, and fusion of these genetic materials encapsulated by EVs mediates the occurrence of HGT (Erdmann et al., 2017). Three main mechanisms underlie HGT: conjugation, transformation, and transduction (Soucy et al., 2015). EVs carrying genetic material may also mediate HGT (Dell’Annunziata et al., 2021). MVs of *Ruminococcus spp* contain double-stranded DNA and promote HGT in bacteria that can catabolize cellulose; thus, wild transformants also have the heritable cellulolytic ability (Klieve et al., 2005). Furthermore, bacteriophages in *B. subtilis* can help MVs perform gene transfer and render the recipient bacterial phage sensitive (Tzipilevich et al., 2016).

Bacterial EVs can resist pressure from the external environment by containing antibiotic-inactivating enzymes; concordantly, bacterial EVs can also use HGT to improve antibiotic resistance; however, most studies focus solely on EVs produced by Gram-negative bacteria (Rumbo et al., 2011; Li et al., 2022). Although HGT-mediated antibiotic resistance exists in Gram-positive bacteria of frequent clinical concern, such as *E. faecalis, S. aureus, Streptococcus, and M. tuberculosis* (Derbyshire and Gray, 2014; Foster, 2017; García-Solache and Rice, 2019), the role of Gram-positive bacterial MVs in HGT, especially in the transfer of resistance genes, remains poorly understood. Therefore, a comprehensive exploration of the association between HGT and MVs could help to address the problem of antibiotic resistance in Gram-positive bacteria.

## 5 Clinical application of Gram-positive bacterial MVs

**Figure 2:**
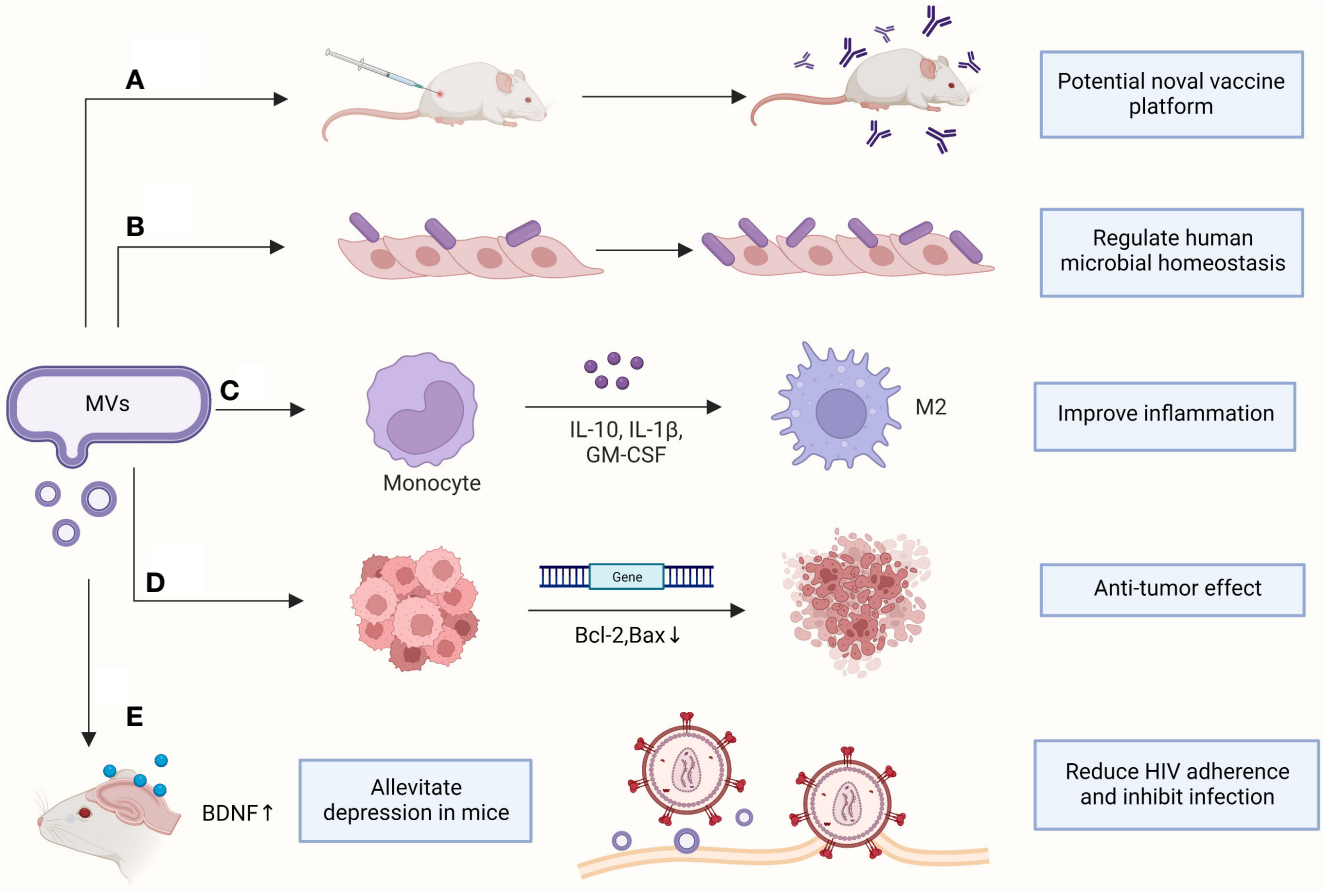
Clinical applications of MVs. **(A)** Antibodies were produced to protect mice from sepsis; **(B)** Increasing the adhesion of probiotics to vaginal epithelia; **(C)** Promoting macrophage polarization and improving inflammatory response; **(D)** Downregulating genes in cancer cells to induce apoptosis; **(E)** Assisting clinical disease treatment. BDNF, brain-derived neurotrophic factor; GM-CSF, Granulocyte-macrophage colony-stimulating factor. (This figure was created using Biorender.com).

Most clinical studies have focused on the hazards of EVs produced by pathogenic bacteria but have neglected the contribution of EVs in diagnosing and treating diseases and maintaining microbial homeostasis in humans (**Figure 2**).

### 5.1 Vaccine preparations

In addition to transporting virulence factors and proteins with immune effects to host cells and inducing inflammatory and immune responses, the immunity and stability of EVs highlights their potential as vaccine candidates. Currently, *S. pneumoniae* and Bacillus Calmette-Guérin (BCG) vaccines are commonly used in clinical practice. However, the capsular polysaccharide vaccine PPSV23 and polysaccharide conjugate vaccine PCV13 in pneumococcal vaccines cover only a limited number of serotypes, and the occurrence of capsular conversion leads to an increase in non-serotype patients. Therefore, there is an urgent need to develop novel vaccines.

Mice vaccinated with nonpathogenic (noncapsular) *S. pneumoniae* MVs have increased survival under live *S. pneumoniae* attack compared with that in unvaccinated mice and provide cross-protection against attacks by different *S. pneumoniae* strains (Choi et al., 2017). Simultaneously, multiple specific immunogenic proteins in *S. pneumoniae* MVs support their potential as next-generation vaccines (Olaya-Abril et al., 2014). Immunization of mice with MVs produced by *S. aureus* mutants with suppression of the alpha toxin-encoding gene resulted in increased levels of cytolysin-neutralizing antibodies in the serum of mice and demonstrated protection in a lethal mouse model of sepsis (Wang et al., 2018) (**Figure 2**).

In addition to their high immunogenicity, MVs have the ability to elicit immune responses in the absence of adjuvants. Vesicles extracted from *M. tuberculosis* can elicit an immune response comparable with that of BCG without an adjuvant and enhance protective efficacy if injected together with BCG (Prados-Rosales et al., 2014). However, the use of MVs as vaccines presents several challenges that need to be addressed. For example, MVs extracted from bacteria contain lipids, toxins, and other substances that can be toxic to humans or result in adverse effects. Moreover, a limited quantity of MVs can be effectively introduced into the human body without undergoing degradation or inducing significant biological responses. Therefore, further investigation and development are required before MV vaccine formulations can pass adequate clinical trials.

### 5.2 Assisting disease diagnosis

The release of probiotic MVs can reflect the relationship between the host microbiome, disease, and health conditions, providing a novel detection technique for diagnosis. MVs in the urine and blood of patients with autism spectrum disorder (ASD) can be used to assess the microbiota of patients and thus understand the role of gut microbiota-brain modulation in ASD symptoms (Lee Y. et al., 2017). The current gold standard for the rapid assessment of tuberculosis is mainly by microbial culture and nucleic acid amplification techniques; however, the long cycle time of microbial culture and poor specificity of the latter often cause delays in diagnosis. Schirmer et al. reported that the immunostrips of vesicles isolated from patients with latent and active tuberculosis were consistently higher than that of healthy volunteers, which may contribute to the diagnosis of latent TB (Schirmer et al., 2022). In addition, pneumococcal MVs isolated from the blood or urine of patients with *Streptococcus pneumoniae*-associated hemolytic uremic syndrome contain highly abundant proteins, which can be used as markers of MVs in Sp-HUS and become the a potential diagnostic indicator for Sp-HUS (Battista et al., 2023).

The use of MVs in the auxiliary diagnosis of diseases is non-invasive and rapid; however, further research must be conducted to determine the accuracy and specificity of MV detection, which would facilitate auxiliary diagnosis of diseases with complex or prolonged diagnoses and deepen our understanding.

### 5.3 Regulating the disorder of human microbial ecology

*Lactobacillus* spp. and *Bifidobacterium* spp. are two commonly used probiotics and Gram-positive bacteria that maintain the healthy state of the human intestine. They are mostly used clinically to regulate intestinal disorders. MVs isolated from *Lactobacillus rhamnosus* JB-1 are involved in the activation and signaling of multiple PRRs between intestinal epithelial cells and the enteric nervous system (Al-Nedawi et al., 2014). In the presence of MVs, *Lactobacillus* enhances host cell adhesion and reduces the adhesion of opportunistic pathogens, contributing to the maintenance of homeostasis in the vagina (Croatti et al., 2022). In addition to probiotics, *Clostridium butyricum* also exhibits a similar effect. *Clostridium butyricum* MVs not only ameliorated symptoms in mice with ulcerative colitis, but also facilitated the restoration of intestinal ecological balance and reconstruction of gut microbiota (Liang et al., 2022).

### 5.4 Improving inflammatory response

In addition to inducing host immune response, MVs can use anti-inflammatory factors to enhance the inflammatory response. For example, *Lactobacillus plantarum* MVs significantly promote the expression of cell surface markers of M2-type macrophages and anti-inflammatory cytokines in human skin tissue cultures, which improve the inflammatory skin state (Kim W. et al., 2020). The blend of *Lactobacillus shortus* MVs and vitamin D3 lessens *H. pylori* attachment to AGS human gastric carcinoma cells, strengthens cell-to-cell connections, and diminishes *H. pylori*-induced inflammation. This combination holds potential as a novel therapy for *H. pylori* infection (Nabavi-Rad et al., 2023).

Some probiotics can improve allergic diseases by inducing antigen-specific regulatory T cells (Tregs). The MVs secreted by these probiotics can exhibit a similar effect. For example, *Bifidobacterium Bifidum* MVs are potential therapeutic adjuvants as they can be used to stimulate *in vitro* dendritic cells to promote Treg cell differentiation and induce proinflammatory factor homeostasis (López et al., 2011). Moreover, MVs produced by *Bifidobacterium longum* contain bacterial extracellular solute-binding protein (ESBP), which reduces the number of mast cells, thereby reducing allergic reactions to food (Kim et al., 2015). *Micrococcus luteus* MV-specific IgG1 and IgG4 levels were significantly lower in asthmatic individuals than in healthy subjects. In asthmatic mice, these MVs decreased IL-1β and IL-17 levels, along with the number of group 3 innate lymphoid cells (ILC3s), thus contributing to asthma alleviation (Sim et al., 2023).

### 5.5 Anti-tumor effect

Currently, the development of cancer treatments is focused on targeted drugs, but other strategies to identify and activate tumor immunity are also being explored. Many bacterial products, such as toxins, peptides, and enzymes, have been increasingly developed for cancer therapy (Soleimani and Javadi, 2022). Some Gram-negative bacterial OMVs have been used as anti-tumor therapeutic vector nanovaccines to inhibit tumor growth based on their potent adjuvant-induced antibody production ability (Huang et al., 2020). Gram-positive bacterial MVs also exhibit properties that could be helpful for the development of cancer treatments. *Lactobacillus rhamnosus* GG is a probiotic strain commonly used as a probiotic supplement. It produces MVs that have cytotoxic effects on cancer cells at certain concentrations and induce apoptosis in liver cancer cells, primarily by downregulating the expression of *bcl-2* and *bax* genes in cancer cells (Behzadi et al., 2017). Similarly, MVs produced by *Lacticaseibacillus paracasei* PC-H1 can penetrate colorectal cancer cells, leading to significant suppression of phosphorylation of 3-phosphoinositide-dependent protein kinase-1 (PDK1) and serine/threonine protein kinase (AKT). This, in turn, downregulates Bcl-2 protein expression, ultimately inducing apoptosis in cancer cells and demonstrating antitumor effects (Shi et al., 2022). Kim et al. found that MVs from *Lactobacillus acidophilus* and *S. aureus* exhibited anti-tumor effects. The lack of an increase in tumor size in mice treated with these MVs may be related to the induction of interferon (INF)-γ production by MV surface proteins (Kim et al., 2017). The development of Gram-positive EVs, primarily comprising probiotics, is emerging as a promising avenue in anticancer therapy. Compared to Gram-negative bacteria, probiotics aid in the restoration of healthy microbiota, ameliorating complications arising from radiation and chemotherapy while enhancing the efficacy of cancer treatment (Li et al., 2021).

### 5.6 Other clinical disease treatment

MVs aid in disease treatment in addition to assisting with diagnosis, contributing to the establishment of a normal ecological environment in the intestinal tract, improving inflammatory responses, and exhibiting anti-tumor effects. The expression of brain-derived neurotrophic factor (BDNF) in the hippocampus increased in depressed mice treated with *L. plantarum* MVs. Depression-like behaviors were alleviated, implying that *L. plantarum* MVs may have an antidepressant effect on neuronal cells (Choi et al., 2019). In both *in vivo* and *in vitro* trials, *Lactobacillus plantarum* MVs treatment notably boosted microRNA-101a-3p expression. Elevating this miRNA distinctly reduced ischemic neuron apoptosis, offering a new approach for ischemic stroke treatment (Yang et al., 2022). MVs released from *Lactobacillus* isolated from the vagina of healthy women help protect human T cells against HIV-1 infection by inhibiting viral attachment and entry into target cells (Ñahui Palomino et al., 2019). In addition, *Lactobacillus druckerii* MVs and *Streptococcus epidermidis* MVs have potential therapeutic value for dermatological issues such as hyperplastic scars and psoriasis, respectively (Gómez-Chávez et al., 2021; Han et al., 2023).

## 6 Summary and outlook

Gram-positive bacteria are a vital class of pathogens that cause infections in humans. With the continuous improvement of bacterial EV extraction technology, many studies have demonstrated that Gram-positive bacteria can produce MVs and that these MVs are closely related to the virulence, immune ability, and pathogenic factors of bacteria. MVs perform many functions, including assisting bacteria in evading host killing, eliciting immune responses, and mediating drug-resistance gene transfer. However, these findings are in the primary research phase, and we lack comprehensive information on the molecular mechanisms of MV production, immune response, and gene transfer. Furthermore, several phenomena and problems remain unsolved.

MVs resist the survival mechanism of external environmental stress, such as antibiotic killing. These findings suggest a novel therapeutic approach to counter the threat of antibiotic resistance in bacteria. In addition, the immune response triggered by MVs highlights their potential as candidates for vaccine development. However, there is no clear experimental evidence regarding the safety of MVs or the development of sequelae when they are injected into humans as a vaccine. These issues require further exploration to expand the application of MVs.

Disparate attention has been paid to the deleterious effects of MVs from clinically pathogenic Gram-positive bacteria while ignoring the beneficial effects of MVs on the human body. Some probiotics, specifically Gram-positive bacteria, exhibit important roles in anti-allergy, anti-inflammatory, and disease suppression responses; these characteristics can be utilized to apply probiotic MVs as oral preparations or pharmaceutical supplements to prevent or treat diseases. Moreover, investigating the role of intestinal probiotic MVs in immunomodulation and signaling will offer a new direction in research of the microbe-gut-brain axis. Therefore, further mechanistic research on MVs is essential, and the roles of several Gram-positive bacteria remain to be explored.

## Author contributions

YX: Supervision, Validation, Writing – original draft, Writing – review and editing. CX: Writing – review and editing. YL: Writing – review and editing. XQ: Writing – review and editing. JL: Supervision, Validation, Visualization, Writing – review and editing.
